# 2-[2-(2-Chloro­phen­yl)-2-oxoeth­yl]-2,3-dihydro-1λ^6^,2-benzothia­zole-1,1,3-trione

**DOI:** 10.1107/S1600536812036653

**Published:** 2012-08-31

**Authors:** Nazia Sattar, Hamid Latif Siddiqui, Naveed Ahmad, Tanvir Hussain, Masood Parvez

**Affiliations:** a Institute of Chemistry, University of the Punjab, Lahore 54590, Pakistan; b Department of Chemistry, The University of Calgary, 2500 University Drive NW, Calgary, Alberta, Canada T2N 1N4

## Abstract

The asymmetric unit of the title compound, C_15_H_10_ClNO_4_S, contains two independent conformers wherein the 2-chloro­phenyl group in one is rotated by approximately 180° compared to the other mol­ecule. This affects the S—N—C—C(=O) and N—C—C(=O)—C torsion angles giving vlaues of −87.0 (2) and 158.7 (2)° in one mol­ecule and −104.3 (2) and −173.4 (2)° in the other. The benzisothia­zole ring systems in the two mol­ecules are essentially planar (r.m.s. deviations = 0.017 and 0.010 Å) and form dihedral angles of 73.53 (7) and 73.26 (6)° with the benzene rings. In the crystal, there are weak π–π inter­actions between the benzene rings of the benzisothia­zole groups and symmetry-related chloro­benzene rings with centroid–centroid distances of 3.6178 (13) and 3.6267 (15) Å. In addition, pairs of weak inter­molecular C—H⋯O hydrogen bonds form inversion dimers which are connected by further C—H⋯O hydrogen bonds into a three-dimensional network.

## Related literature

For the bromo-substituted analog of the title compound, see: Sattar *et al.* (2012 ▶). For related structures, see: Maliha *et al.* (2007 ▶); Siddiqui *et al.* (2007 ▶).
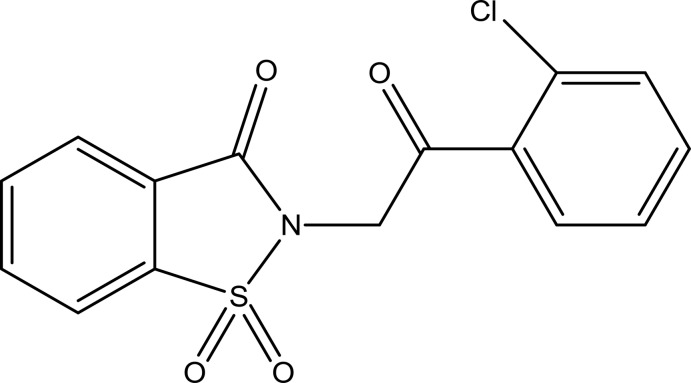


## Experimental

### 

#### Crystal data

C_15_H_10_ClNO_4_S
*M* *_r_* = 335.75
Triclinic, 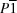

*a* = 7.4933 (2) Å
*b* = 13.9702 (3) Å
*c* = 14.5844 (3) Å
α = 109.0462 (14)°
β = 96.5998 (14)°
γ = 93.4671 (11)°
*V* = 1425.77 (6) Å^3^
*Z* = 4
Mo *K*α radiation
μ = 0.43 mm^−1^
*T* = 123 K
0.16 × 0.14 × 0.10 mm

#### Data collection

Nonius KappaCCD diffractometer
Absorption correction: multi-scan (*SORTAV*; Blessing, 1997 ▶) *T*_min_ = 0.934, *T*_max_ = 0.958
12676 measured reflections
6602 independent reflections
5465 reflections with *I* > 2σ(*I*)
*R*_int_ = 0.031

#### Refinement

*R*[*F* ^2^ > 2σ(*F* ^2^)] = 0.045
*wR*(*F* ^2^) = 0.105
*S* = 1.03
6602 reflections
397 parameters
H-atom parameters constrained
Δρ_max_ = 0.34 e Å^−3^
Δρ_min_ = −0.46 e Å^−3^

### 

Data collection: *COLLECT* (Hooft, 1998 ▶); cell refinement: *DENZO* (Otwinowski & Minor, 1997 ▶); data reduction: *SCALEPACK* (Otwinowski & Minor, 1997 ▶); program(s) used to solve structure: *SHELXS97* (Sheldrick, 2008 ▶); program(s) used to refine structure: *SHELXL97* (Sheldrick, 2008 ▶); molecular graphics: *ORTEP-3 for Windows* (Farrugia, 1997 ▶); software used to prepare material for publication: *SHELXL97*.

## Supplementary Material

Crystal structure: contains datablock(s) global, I. DOI: 10.1107/S1600536812036653/lh5517sup1.cif

Structure factors: contains datablock(s) I. DOI: 10.1107/S1600536812036653/lh5517Isup2.hkl

Supplementary material file. DOI: 10.1107/S1600536812036653/lh5517Isup3.cml

Additional supplementary materials:  crystallographic information; 3D view; checkCIF report

## Figures and Tables

**Table 1:** Hydrogen-bond geometry (Å, °)

*D*—H⋯*A*	*D*—H	H⋯*A*	*D*⋯*A*	*D*—H⋯*A*
C3—H3⋯O7^i^	0.95	2.53	3.234 (3)	131
C14—H14⋯O1^ii^	0.95	2.39	3.284 (3)	158
C17—H17⋯O5^iii^	0.95	2.43	3.213 (3)	139
C27—H27⋯O7^iv^	0.95	2.27	3.133 (3)	151
C30—H30⋯O2^v^	0.95	2.51	3.219 (3)	132

Symmetry codes: (i) 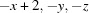; (ii) 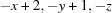; (iii) 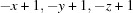; (iv) 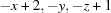; (v) 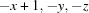.

**Fig. 1.**
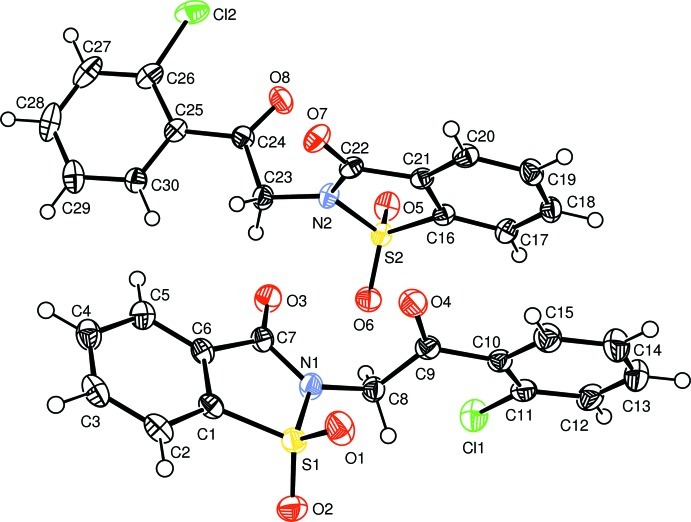
The asymmetric unit of the title compound with displacement ellipsoids drawn at the 50% probability level. H atoms are presented as small spheres of arbitrary radius.

**Fig. 2.**
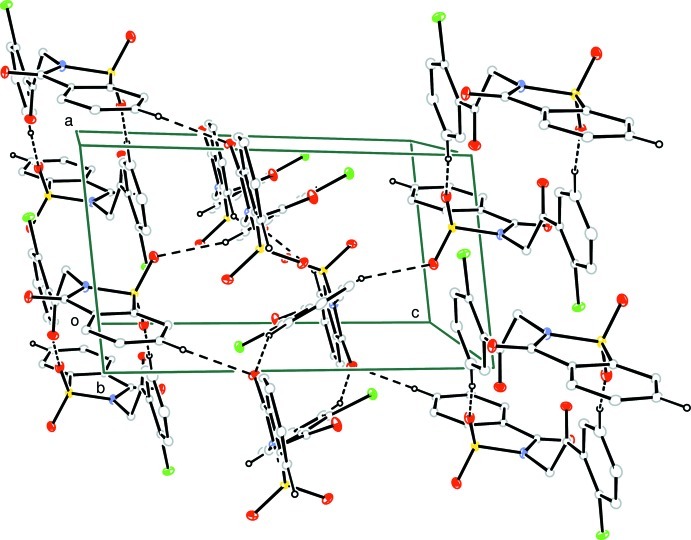
Part of the crystal structure with C—H···O hydrogen bonds shown as dashed lines. H atoms non-participating in hydrogen-bonding are omitted for clarity.

## supplementary crystallographic information

### Comment

The crystal structure of the bromoisomorph of the title molecule has been reported by our research group recently (Sattar *et al.*, (2012). In this article we report the synthesis and crystal structure of the title compound.

The asymmetric unit of the title compound contains two conformers (Fig. 1). In both molecules, the benzisothiazol rings S1/N1/C1–C7 and S2/N2/C16–C22 are essentially planar with r.m.s. deviations of fitted atoms being 0.017 and 0.010 Å, respectively, while the mean-planes of the benzene rings C10–C15 and C25–C30 form dihedral angles 73.53 (7) and 73.26 (6)°, respectively, with the mean-planes of the benzisothiazole ring systems. The orientation of the Cl atoms in the two conformers exhibit the most pronounced difference, with opposing orientations in the two molecules. The crystal structure is stabilized by π–π interactions between benzene rings (C1–C6) of the benzisothiazole moities in one molecule and chlorobenzene rings (C25–C30) in a symmetry related molecule centroid to centroid distances of 3.6168 (13) and 3.62672 (15) Å. The crystal packing is further consolidated by weak intermolecular C—H···O hydrogen bonds. The molecule containing S1 forms centrosymmetric dimers *via* C14—H14···O1^ii^ hydrogen bonding interactions. The other molecule also forms centrosymmetric dimers *via* C17—H17···O5^iii^ hydrogen bonds. Futher hydrogen bonding interactions of the type C—H···O result in a 3-D network (Fig. 2 and Tab. 1).

The bond distances and angles in both molecules of the title compound agree very well with the corresponding bond distances and angles reported in closely related compounds (Sattar *et al.*, (2012); Maliha *et al.*, 2007; Siddiqui *et al.*, 2007).

### Experimental

A mixture of 2-chloro-1-(2-chlorophenyl)ethanone (1.62 g, 8.56 mmol), sodium saccharine (2.1 g, 10.3 mmol) and dimethylformamide (15 mL) was stirred at 383 K for a period of 3 hours under anhydrous conditions. The reaction mixture was cooled to room temperature and transferred to ice cooled water. The pale yellow precipitate of the title compound formed, were filtered and washed with water and cold ethanol, respectively. The crystals suitable for diffraction were grown from a solution of the title compound EtOAc-CHCl_3_ (1:1) by slow evaporation. Yield = 2.19 g, 76%; 385–387 K.

### Refinement

All H atoms were positioned geometrically and refined using a riding model, with C—H = 0.95 and 0.99 Å, for aryl and methylene H-atoms, respectively. The *U*_iso_(H) were allowed at 1.2*U*_eq_(C).

### Crystal data

**Table:**

C_15_H_10_ClNO_4_S	*Z* = 4
*M**_r_* = 335.75	*F*(000) = 688
Triclinic, *P*1	*D*_x_ = 1.564 Mg m^−^^3^
Hall symbol: -P 1	Mo *K*α radiation, λ = 0.71073 Å
*a* = 7.4933 (2) Å	Cell parameters from 6513 reflections
*b* = 13.9702 (3) Å	θ = 1.0–27.5°
*c* = 14.5844 (3) Å	µ = 0.43 mm^−^^1^
α = 109.0462 (14)°	*T* = 123 K
β = 96.5998 (14)°	Block, colorless
γ = 93.4671 (11)°	0.16 × 0.14 × 0.10 mm
*V* = 1425.77 (6) Å^3^	

### Data collection

**Table:**

Nonius KappaCCD diffractometer	6602 independent reflections
Radiation source: fine-focus sealed tube	5465 reflections with *I* > 2σ(*I*)
Graphite monochromator	*R*_int_ = 0.031
ω and φ scans	θ_max_ = 27.7°, θ_min_ = 2.8°
Absorption correction: multi-scan (*SORTAV*; Blessing, 1997)	*h* = −9→9
*T*_min_ = 0.934, *T*_max_ = 0.958	*k* = −18→18
12676 measured reflections	*l* = −18→19

### Refinement

**Table:**

Refinement on *F*^2^	Primary atom site location: structure-invariant direct methods
Least-squares matrix: full	Secondary atom site location: difference Fourier map
*R*[*F*^2^ > 2σ(*F*^2^)] = 0.045	Hydrogen site location: inferred from neighbouring sites
*wR*(*F*^2^) = 0.105	H-atom parameters constrained
*S* = 1.03	*w* = 1/[σ^2^(*F*_o_^2^) + (0.0289*P*)^2^ + 1.9353*P*] where *P* = (*F*_o_^2^ + 2*F*_c_^2^)/3
6602 reflections	(Δ/σ)_max_ < 0.001
397 parameters	Δρ_max_ = 0.34 e Å^−^^3^
0 restraints	Δρ_min_ = −0.46 e Å^−^^3^

### Special details

**Table:**

Geometry. All esds (except the esd in the dihedral angle between two l.s. planes) are estimated using the full covariance matrix. The cell esds are taken into account individually in the estimation of esds in distances, angles and torsion angles; correlations between esds in cell parameters are only used when they are defined by crystal symmetry. An approximate (isotropic) treatment of cell esds is used for estimating esds involving l.s. planes.
Refinement. Refinement of *F*^2^ against ALL reflections. The weighted *R*-factor *wR* and goodness of fit *S* are based on *F*^2^, conventional *R*-factors *R* are based on *F*, with *F* set to zero for negative *F*^2^. The threshold expression of *F*^2^ > σ(*F*^2^) is used only for calculating *R*-factors(gt) *etc*. and is not relevant to the choice of reflections for refinement. *R*-factors based on *F*^2^ are statistically about twice as large as those based on *F*, and *R*- factors based on ALL data will be even larger.

### Fractional atomic coordinates and isotropic or equivalent isotropic displacement parameters (Å^2^)

**Table:**

	*x*	*y*	*z*	*U*_iso_*/*U*_eq_	
Cl1	0.37851 (8)	0.45393 (5)	0.14792 (5)	0.03709 (15)	
Cl2	0.91058 (10)	0.08491 (6)	0.65590 (4)	0.04241 (17)	
S1	0.67930 (8)	0.13106 (4)	−0.09566 (4)	0.02600 (13)	
S2	0.60770 (7)	0.34957 (4)	0.38890 (4)	0.02241 (12)	
O1	0.8259 (3)	0.18938 (14)	−0.11376 (13)	0.0381 (4)	
O2	0.5083 (2)	0.12158 (13)	−0.15434 (12)	0.0348 (4)	
O3	0.6914 (2)	0.13362 (13)	0.16209 (11)	0.0315 (4)	
O4	0.9088 (2)	0.33337 (13)	0.11577 (13)	0.0343 (4)	
O5	0.5508 (2)	0.37794 (13)	0.48340 (12)	0.0317 (4)	
O6	0.4695 (2)	0.31718 (13)	0.30561 (12)	0.0312 (4)	
O7	1.0402 (2)	0.23212 (13)	0.35123 (12)	0.0301 (4)	
O8	0.8149 (3)	0.21186 (13)	0.54195 (13)	0.0400 (5)	
N1	0.6544 (3)	0.17557 (14)	0.02268 (13)	0.0263 (4)	
N2	0.7514 (3)	0.26105 (14)	0.37907 (14)	0.0237 (4)	
C1	0.7391 (3)	0.01406 (17)	−0.08817 (16)	0.0239 (4)	
C2	0.7780 (3)	−0.06900 (18)	−0.16356 (17)	0.0288 (5)	
H2	0.7729	−0.0684	−0.2288	0.035*	
C3	0.8247 (3)	−0.15304 (18)	−0.13889 (18)	0.0306 (5)	
H3	0.8507	−0.2119	−0.1886	0.037*	
C4	0.8343 (3)	−0.15291 (18)	−0.04349 (18)	0.0297 (5)	
H4	0.8678	−0.2114	−0.0289	0.036*	
C5	0.7956 (3)	−0.06863 (17)	0.03141 (17)	0.0266 (5)	
H5	0.8029	−0.0686	0.0969	0.032*	
C6	0.7463 (3)	0.01491 (16)	0.00760 (15)	0.0219 (4)	
C7	0.6976 (3)	0.11188 (16)	0.07564 (16)	0.0233 (4)	
C8	0.5978 (3)	0.27629 (16)	0.06608 (16)	0.0249 (5)	
H8A	0.5333	0.2771	0.1219	0.030*	
H8B	0.5128	0.2918	0.0169	0.030*	
C9	0.7584 (3)	0.35782 (17)	0.10189 (15)	0.0240 (4)	
C10	0.7338 (3)	0.46790 (17)	0.11690 (15)	0.0231 (4)	
C11	0.5777 (3)	0.51623 (18)	0.13554 (16)	0.0261 (5)	
C12	0.5772 (4)	0.61965 (19)	0.15012 (18)	0.0348 (6)	
H12	0.4701	0.6516	0.1631	0.042*	
C13	0.7312 (4)	0.6758 (2)	0.1458 (2)	0.0410 (6)	
H13	0.7303	0.7465	0.1561	0.049*	
C14	0.8871 (4)	0.6298 (2)	0.1265 (2)	0.0402 (6)	
H14	0.9931	0.6684	0.1227	0.048*	
C15	0.8875 (3)	0.52734 (19)	0.11265 (18)	0.0321 (5)	
H15	0.9955	0.4962	0.0999	0.038*	
C16	0.7734 (3)	0.43841 (16)	0.37899 (15)	0.0202 (4)	
C17	0.7539 (3)	0.53697 (17)	0.38192 (16)	0.0240 (4)	
H17	0.6420	0.5649	0.3902	0.029*	
C18	0.9051 (3)	0.59344 (17)	0.37227 (16)	0.0259 (5)	
H18	0.8970	0.6616	0.3741	0.031*	
C19	1.0687 (3)	0.55175 (17)	0.35987 (16)	0.0266 (5)	
H19	1.1703	0.5921	0.3536	0.032*	
C20	1.0857 (3)	0.45252 (17)	0.35657 (16)	0.0244 (4)	
H20	1.1972	0.4242	0.3478	0.029*	
C21	0.9355 (3)	0.39583 (16)	0.36650 (15)	0.0207 (4)	
C22	0.9235 (3)	0.28879 (17)	0.36418 (15)	0.0219 (4)	
C23	0.6939 (3)	0.15852 (16)	0.37356 (16)	0.0252 (5)	
H23A	0.7474	0.1096	0.3208	0.030*	
H23B	0.5609	0.1457	0.3567	0.030*	
C24	0.7508 (3)	0.14107 (17)	0.47071 (16)	0.0238 (4)	
C25	0.7229 (3)	0.03392 (17)	0.46952 (16)	0.0224 (4)	
C26	0.7910 (3)	0.00201 (19)	0.54732 (17)	0.0286 (5)	
C27	0.7632 (4)	−0.0988 (2)	0.5410 (2)	0.0373 (6)	
H27	0.8137	−0.1197	0.5933	0.045*	
C28	0.6623 (4)	−0.16928 (19)	0.4590 (2)	0.0395 (7)	
H28	0.6417	−0.2381	0.4558	0.047*	
C29	0.5912 (4)	−0.14066 (18)	0.3819 (2)	0.0340 (6)	
H29	0.5207	−0.1891	0.3258	0.041*	
C30	0.6236 (3)	−0.04047 (17)	0.38706 (17)	0.0257 (5)	
H30	0.5771	−0.0214	0.3329	0.031*	

### Atomic displacement parameters (Å^2^)

**Table:**

	*U*^11^	*U*^22^	*U*^33^	*U*^12^	*U*^13^	*U*^23^
Cl1	0.0276 (3)	0.0352 (3)	0.0512 (4)	0.0092 (2)	0.0128 (3)	0.0148 (3)
Cl2	0.0452 (4)	0.0587 (4)	0.0250 (3)	0.0151 (3)	0.0001 (3)	0.0163 (3)
S1	0.0341 (3)	0.0239 (3)	0.0193 (3)	−0.0003 (2)	0.0034 (2)	0.0071 (2)
S2	0.0216 (3)	0.0217 (3)	0.0266 (3)	0.0037 (2)	0.0076 (2)	0.0101 (2)
O1	0.0518 (12)	0.0333 (10)	0.0296 (9)	−0.0081 (8)	0.0095 (8)	0.0120 (8)
O2	0.0414 (10)	0.0362 (10)	0.0255 (8)	0.0069 (8)	−0.0013 (7)	0.0102 (7)
O3	0.0454 (10)	0.0299 (9)	0.0207 (8)	0.0082 (8)	0.0094 (7)	0.0083 (7)
O4	0.0266 (9)	0.0289 (9)	0.0423 (10)	0.0077 (7)	−0.0005 (7)	0.0059 (8)
O5	0.0330 (9)	0.0327 (9)	0.0346 (9)	0.0068 (7)	0.0168 (7)	0.0138 (7)
O6	0.0233 (8)	0.0318 (9)	0.0373 (9)	0.0018 (7)	0.0012 (7)	0.0113 (7)
O7	0.0281 (9)	0.0317 (9)	0.0375 (9)	0.0125 (7)	0.0092 (7)	0.0182 (7)
O8	0.0580 (12)	0.0272 (9)	0.0288 (9)	−0.0103 (8)	−0.0076 (8)	0.0084 (7)
N1	0.0367 (11)	0.0217 (9)	0.0204 (9)	0.0044 (8)	0.0059 (8)	0.0062 (7)
N2	0.0260 (10)	0.0205 (9)	0.0287 (10)	0.0042 (7)	0.0076 (8)	0.0124 (8)
C1	0.0216 (11)	0.0230 (11)	0.0245 (11)	−0.0026 (8)	0.0027 (8)	0.0057 (9)
C2	0.0274 (12)	0.0285 (12)	0.0238 (11)	−0.0042 (9)	0.0042 (9)	0.0008 (9)
C3	0.0235 (11)	0.0243 (11)	0.0355 (13)	−0.0009 (9)	0.0077 (10)	−0.0019 (10)
C4	0.0242 (11)	0.0235 (11)	0.0397 (13)	0.0003 (9)	0.0070 (10)	0.0081 (10)
C5	0.0257 (11)	0.0253 (11)	0.0299 (12)	0.0016 (9)	0.0078 (9)	0.0095 (9)
C6	0.0206 (10)	0.0204 (10)	0.0233 (10)	−0.0012 (8)	0.0062 (8)	0.0051 (8)
C7	0.0233 (11)	0.0213 (10)	0.0252 (11)	0.0002 (8)	0.0047 (8)	0.0078 (9)
C8	0.0280 (12)	0.0216 (11)	0.0247 (11)	0.0036 (9)	0.0037 (9)	0.0072 (9)
C9	0.0269 (11)	0.0248 (11)	0.0196 (10)	0.0048 (9)	0.0033 (8)	0.0060 (9)
C10	0.0261 (11)	0.0243 (11)	0.0187 (10)	0.0036 (9)	0.0024 (8)	0.0070 (8)
C11	0.0270 (12)	0.0285 (12)	0.0225 (11)	0.0057 (9)	0.0018 (9)	0.0084 (9)
C12	0.0416 (15)	0.0287 (12)	0.0343 (13)	0.0128 (11)	0.0015 (11)	0.0107 (10)
C13	0.0545 (18)	0.0247 (12)	0.0429 (15)	0.0028 (12)	0.0007 (13)	0.0125 (11)
C14	0.0447 (16)	0.0324 (14)	0.0445 (15)	−0.0070 (12)	0.0035 (12)	0.0169 (12)
C15	0.0320 (13)	0.0318 (13)	0.0328 (13)	0.0007 (10)	0.0050 (10)	0.0117 (10)
C16	0.0205 (10)	0.0233 (10)	0.0191 (10)	0.0022 (8)	0.0049 (8)	0.0098 (8)
C17	0.0258 (11)	0.0237 (11)	0.0247 (11)	0.0060 (9)	0.0072 (9)	0.0091 (9)
C18	0.0341 (12)	0.0189 (10)	0.0241 (11)	0.0020 (9)	0.0051 (9)	0.0064 (9)
C19	0.0282 (12)	0.0257 (11)	0.0263 (11)	−0.0035 (9)	0.0047 (9)	0.0100 (9)
C20	0.0186 (10)	0.0296 (12)	0.0263 (11)	0.0035 (9)	0.0036 (8)	0.0108 (9)
C21	0.0219 (10)	0.0235 (10)	0.0177 (10)	0.0040 (8)	0.0032 (8)	0.0077 (8)
C22	0.0227 (11)	0.0259 (11)	0.0201 (10)	0.0053 (9)	0.0040 (8)	0.0109 (8)
C23	0.0317 (12)	0.0206 (10)	0.0249 (11)	0.0020 (9)	0.0049 (9)	0.0096 (9)
C24	0.0246 (11)	0.0246 (11)	0.0235 (11)	−0.0003 (9)	0.0047 (9)	0.0098 (9)
C25	0.0218 (11)	0.0257 (11)	0.0244 (11)	0.0055 (9)	0.0088 (8)	0.0122 (9)
C26	0.0290 (12)	0.0369 (13)	0.0273 (11)	0.0141 (10)	0.0126 (9)	0.0161 (10)
C27	0.0433 (15)	0.0456 (15)	0.0419 (14)	0.0260 (13)	0.0238 (12)	0.0306 (13)
C28	0.0507 (17)	0.0251 (12)	0.0568 (17)	0.0160 (12)	0.0338 (14)	0.0217 (12)
C29	0.0364 (14)	0.0215 (11)	0.0448 (15)	0.0034 (10)	0.0185 (11)	0.0078 (10)
C30	0.0288 (12)	0.0232 (11)	0.0267 (11)	0.0034 (9)	0.0071 (9)	0.0092 (9)

### Geometric parameters (Å, º)

**Table:**

Cl1—C11	1.739 (2)	C10—C15	1.398 (3)
Cl2—C26	1.733 (3)	C11—C12	1.390 (3)
S1—O1	1.4277 (18)	C12—C13	1.375 (4)
S1—O2	1.4300 (18)	C12—H12	0.9500
S1—N1	1.6697 (19)	C13—C14	1.380 (4)
S1—C1	1.754 (2)	C13—H13	0.9500
S2—O5	1.4273 (17)	C14—C15	1.379 (4)
S2—O6	1.4310 (17)	C14—H14	0.9500
S2—N2	1.6700 (19)	C15—H15	0.9500
S2—C16	1.754 (2)	C16—C17	1.381 (3)
O3—C7	1.204 (3)	C16—C21	1.389 (3)
O4—C9	1.211 (3)	C17—C18	1.388 (3)
O7—C22	1.205 (3)	C17—H17	0.9500
O8—C24	1.202 (3)	C18—C19	1.394 (3)
N1—C7	1.385 (3)	C18—H18	0.9500
N1—C8	1.454 (3)	C19—C20	1.385 (3)
N2—C22	1.385 (3)	C19—H19	0.9500
N2—C23	1.444 (3)	C20—C21	1.386 (3)
C1—C2	1.386 (3)	C20—H20	0.9500
C1—C6	1.388 (3)	C21—C22	1.482 (3)
C2—C3	1.388 (4)	C23—C24	1.532 (3)
C2—H2	0.9500	C23—H23A	0.9900
C3—C4	1.384 (4)	C23—H23B	0.9900
C3—H3	0.9500	C24—C25	1.493 (3)
C4—C5	1.392 (3)	C25—C30	1.402 (3)
C4—H4	0.9500	C25—C26	1.404 (3)
C5—C6	1.381 (3)	C26—C27	1.383 (4)
C5—H5	0.9500	C27—C28	1.380 (4)
C6—C7	1.490 (3)	C27—H27	0.9500
C8—C9	1.523 (3)	C28—C29	1.374 (4)
C8—H8A	0.9900	C28—H28	0.9500
C8—H8B	0.9900	C29—C30	1.382 (3)
C9—C10	1.507 (3)	C29—H29	0.9500
C10—C11	1.396 (3)	C30—H30	0.9500
			
O1—S1—O2	117.15 (11)	C12—C13—C14	120.2 (2)
O1—S1—N1	109.99 (10)	C12—C13—H13	119.9
O2—S1—N1	109.32 (10)	C14—C13—H13	119.9
O1—S1—C1	112.33 (11)	C15—C14—C13	119.4 (3)
O2—S1—C1	112.65 (11)	C15—C14—H14	120.3
N1—S1—C1	92.64 (10)	C13—C14—H14	120.3
O5—S2—O6	117.21 (11)	C14—C15—C10	121.9 (2)
O5—S2—N2	109.79 (10)	C14—C15—H15	119.0
O6—S2—N2	109.74 (10)	C10—C15—H15	119.0
O5—S2—C16	112.99 (10)	C17—C16—C21	122.5 (2)
O6—S2—C16	111.84 (10)	C17—C16—S2	127.32 (17)
N2—S2—C16	92.49 (10)	C21—C16—S2	110.21 (16)
C7—N1—C8	123.36 (18)	C16—C17—C18	117.0 (2)
C7—N1—S1	115.46 (15)	C16—C17—H17	121.5
C8—N1—S1	121.14 (15)	C18—C17—H17	121.5
C22—N2—C23	122.02 (18)	C17—C18—C19	121.1 (2)
C22—N2—S2	115.33 (14)	C17—C18—H18	119.5
C23—N2—S2	122.17 (16)	C19—C18—H18	119.5
C2—C1—C6	122.6 (2)	C20—C19—C18	121.1 (2)
C2—C1—S1	127.34 (18)	C20—C19—H19	119.4
C6—C1—S1	110.04 (16)	C18—C19—H19	119.4
C1—C2—C3	116.6 (2)	C19—C20—C21	118.1 (2)
C1—C2—H2	121.7	C19—C20—H20	121.0
C3—C2—H2	121.7	C21—C20—H20	121.0
C4—C3—C2	121.4 (2)	C20—C21—C16	120.2 (2)
C4—C3—H3	119.3	C20—C21—C22	126.94 (19)
C2—C3—H3	119.3	C16—C21—C22	112.88 (19)
C3—C4—C5	121.2 (2)	O7—C22—N2	123.5 (2)
C3—C4—H4	119.4	O7—C22—C21	127.5 (2)
C5—C4—H4	119.4	N2—C22—C21	109.01 (18)
C6—C5—C4	117.9 (2)	N2—C23—C24	111.54 (18)
C6—C5—H5	121.0	N2—C23—H23A	109.3
C4—C5—H5	121.0	C24—C23—H23A	109.3
C5—C6—C1	120.2 (2)	N2—C23—H23B	109.3
C5—C6—C7	126.7 (2)	C24—C23—H23B	109.3
C1—C6—C7	113.05 (19)	H23A—C23—H23B	108.0
O3—C7—N1	123.8 (2)	O8—C24—C25	124.0 (2)
O3—C7—C6	127.5 (2)	O8—C24—C23	119.7 (2)
N1—C7—C6	108.69 (18)	C25—C24—C23	116.26 (18)
N1—C8—C9	111.51 (18)	C30—C25—C26	116.9 (2)
N1—C8—H8A	109.3	C30—C25—C24	119.42 (19)
C9—C8—H8A	109.3	C26—C25—C24	123.6 (2)
N1—C8—H8B	109.3	C27—C26—C25	120.8 (2)
C9—C8—H8B	109.3	C27—C26—Cl2	116.34 (19)
H8A—C8—H8B	108.0	C25—C26—Cl2	122.81 (19)
O4—C9—C10	119.5 (2)	C28—C27—C26	120.3 (2)
O4—C9—C8	119.5 (2)	C28—C27—H27	119.9
C10—C9—C8	121.01 (19)	C26—C27—H27	119.9
C11—C10—C15	117.4 (2)	C29—C28—C27	120.6 (2)
C11—C10—C9	127.5 (2)	C29—C28—H28	119.7
C15—C10—C9	115.1 (2)	C27—C28—H28	119.7
C12—C11—C10	120.8 (2)	C28—C29—C30	119.1 (2)
C12—C11—Cl1	116.29 (19)	C28—C29—H29	120.4
C10—C11—Cl1	122.82 (18)	C30—C29—H29	120.4
C13—C12—C11	120.2 (2)	C29—C30—C25	122.2 (2)
C13—C12—H12	119.9	C29—C30—H30	118.9
C11—C12—H12	119.9	C25—C30—H30	118.9
			
O1—S1—N1—C7	−111.31 (18)	Cl1—C11—C12—C13	177.3 (2)
O2—S1—N1—C7	118.75 (18)	C11—C12—C13—C14	0.3 (4)
C1—S1—N1—C7	3.62 (18)	C12—C13—C14—C15	−0.7 (4)
O1—S1—N1—C8	66.4 (2)	C13—C14—C15—C10	0.5 (4)
O2—S1—N1—C8	−63.5 (2)	C11—C10—C15—C14	0.2 (3)
C1—S1—N1—C8	−178.64 (18)	C9—C10—C15—C14	−179.0 (2)
O5—S2—N2—C22	−118.06 (16)	O5—S2—C16—C17	−66.6 (2)
O6—S2—N2—C22	111.76 (17)	O6—S2—C16—C17	68.2 (2)
C16—S2—N2—C22	−2.52 (17)	N2—S2—C16—C17	−179.3 (2)
O5—S2—N2—C23	69.74 (19)	O5—S2—C16—C21	113.82 (16)
O6—S2—N2—C23	−60.45 (19)	O6—S2—C16—C21	−111.35 (16)
C16—S2—N2—C23	−174.73 (17)	N2—S2—C16—C21	1.08 (16)
O1—S1—C1—C2	−68.9 (2)	C21—C16—C17—C18	−0.3 (3)
O2—S1—C1—C2	66.0 (2)	S2—C16—C17—C18	−179.87 (17)
N1—S1—C1—C2	178.2 (2)	C16—C17—C18—C19	0.2 (3)
O1—S1—C1—C6	110.06 (17)	C17—C18—C19—C20	0.1 (3)
O2—S1—C1—C6	−115.04 (17)	C18—C19—C20—C21	−0.3 (3)
N1—S1—C1—C6	−2.83 (17)	C19—C20—C21—C16	0.2 (3)
C6—C1—C2—C3	0.2 (3)	C19—C20—C21—C22	179.4 (2)
S1—C1—C2—C3	179.07 (18)	C17—C16—C21—C20	0.1 (3)
C1—C2—C3—C4	−0.9 (3)	S2—C16—C21—C20	179.73 (16)
C2—C3—C4—C5	0.6 (4)	C17—C16—C21—C22	−179.13 (19)
C3—C4—C5—C6	0.3 (3)	S2—C16—C21—C22	0.5 (2)
C4—C5—C6—C1	−1.0 (3)	C23—N2—C22—O7	−4.3 (3)
C4—C5—C6—C7	179.3 (2)	S2—N2—C22—O7	−176.55 (18)
C2—C1—C6—C5	0.8 (3)	C23—N2—C22—C21	175.33 (18)
S1—C1—C6—C5	−178.28 (17)	S2—N2—C22—C21	3.1 (2)
C2—C1—C6—C7	−179.5 (2)	C20—C21—C22—O7	−1.8 (4)
S1—C1—C6—C7	1.5 (2)	C16—C21—C22—O7	177.4 (2)
C8—N1—C7—O3	0.4 (4)	C20—C21—C22—N2	178.6 (2)
S1—N1—C7—O3	178.06 (19)	C16—C21—C22—N2	−2.2 (2)
C8—N1—C7—C6	179.05 (19)	C22—N2—C23—C24	84.0 (2)
S1—N1—C7—C6	−3.3 (2)	S2—N2—C23—C24	−104.3 (2)
C5—C6—C7—O3	−0.7 (4)	N2—C23—C24—O8	8.2 (3)
C1—C6—C7—O3	179.6 (2)	N2—C23—C24—C25	−171.35 (18)
C5—C6—C7—N1	−179.3 (2)	O8—C24—C25—C30	170.8 (2)
C1—C6—C7—N1	1.0 (3)	C23—C24—C25—C30	−9.7 (3)
C7—N1—C8—C9	90.5 (3)	O8—C24—C25—C26	−9.2 (4)
S1—N1—C8—C9	−87.0 (2)	C23—C24—C25—C26	170.3 (2)
N1—C8—C9—O4	−19.1 (3)	C30—C25—C26—C27	1.2 (3)
N1—C8—C9—C10	158.73 (19)	C24—C25—C26—C27	−178.8 (2)
O4—C9—C10—C11	−157.7 (2)	C30—C25—C26—Cl2	−178.03 (17)
C8—C9—C10—C11	24.5 (3)	C24—C25—C26—Cl2	2.0 (3)
O4—C9—C10—C15	21.4 (3)	C25—C26—C27—C28	−2.2 (4)
C8—C9—C10—C15	−156.5 (2)	Cl2—C26—C27—C28	177.04 (19)
C15—C10—C11—C12	−0.6 (3)	C26—C27—C28—C29	1.3 (4)
C9—C10—C11—C12	178.5 (2)	C27—C28—C29—C30	0.7 (4)
C15—C10—C11—Cl1	−177.34 (17)	C28—C29—C30—C25	−1.7 (4)
C9—C10—C11—Cl1	1.7 (3)	C26—C25—C30—C29	0.8 (3)
C10—C11—C12—C13	0.4 (4)	C24—C25—C30—C29	−179.2 (2)

### Hydrogen-bond geometry (Å, º)

**Table:**

*D*—H···*A*	*D*—H	H···*A*	*D*···*A*	*D*—H···*A*
C3—H3···O7^i^	0.95	2.53	3.234 (3)	131
C14—H14···O1^ii^	0.95	2.39	3.284 (3)	158
C17—H17···O5^iii^	0.95	2.43	3.213 (3)	139
C27—H27···O7^iv^	0.95	2.27	3.133 (3)	151
C30—H30···O2^v^	0.95	2.51	3.219 (3)	132

Symmetry codes: (i) −*x*+2, −*y*, −*z*; (ii) −*x*+2, −*y*+1, −*z*; (iii) −*x*+1, −*y*+1, −*z*+1; (iv) −*x*+2, −*y*, −*z*+1; (v) −*x*+1, −*y*, −*z*.
